# Human Epididymis Protein 4 Promotes Events Associated with Metastatic Ovarian Cancer *via* Regulation of the Extracelluar Matrix

**DOI:** 10.3389/fonc.2017.00332

**Published:** 2018-01-22

**Authors:** Jennifer R. Ribeiro, Hilary M. Gaudet, Mehreen Khan, Christoph Schorl, Nicole E. James, Matthew T. Oliver, Paul A. DiSilvestro, Richard G. Moore, Naohiro Yano

**Affiliations:** ^1^Division of Gynecologic Oncology, Department of Obstetrics and Gynecology, Program in Women’s Oncology, Women and Infants Hospital, Providence, RI, United States; ^2^Department of Chemistry, Wheaton College, Norton, MA, United States; ^3^Center for Genomics and Proteomics, Genomics Core Facility, Brown University, Providence, RI, United States; ^4^Department of Obstetrics and Gynecology, Wilmot Cancer Institute, Division of Gynecologic Oncology, University of Rochester Medical Center, Rochester, NY, United States; ^5^Roger Williams Medical Center, Department of Surgery, Boston University Medical School, Providence, RI, United States; ^6^Department of Biomedical and Pharmaceutical Sciences, University of Rhode Island, Kingston, RI, United States

**Keywords:** human epididymis protein 4, epithelial ovarian cancer, metastasis, invasion, haptotaxis, adhesion, protein kinase signaling, OVCAR8 cells

## Abstract

Human epididymis protein 4 (HE4) has received much attention recently due to its diagnostic and prognostic abilities for epithelial ovarian cancer. Since its inclusion in the Risk of Ovarian Malignancy Algorithm (ROMA), studies have focused on its functional effects in ovarian cancer. Here, we aimed to investigate the role of HE4 in invasion, haptotaxis, and adhesion of ovarian cancer cells. Furthermore, we sought to gain an understanding of relevant transcriptional profiles and protein kinase signaling pathways mediated by this multifunctional protein. Exposure of OVCAR8 ovarian cancer cells to recombinant HE4 (rHE4) promoted invasion, haptotaxis toward a fibronectin substrate, and adhesion onto fibronectin. Overexpression of HE4 or treatment with rHE4 led to upregulation of several transcripts coding for extracellular matrix proteins, including *SERPINB2, GREM1, LAMC2*, and *LAMB3*. Gene ontology indicated an enrichment of terms related to extracellular matrix, cell migration, adhesion, growth, and kinase phosphorylation. LAMC2 and LAMB3 protein levels were constitutively elevated in cells overexpressing HE4 and were upregulated in a time-dependent manner in cells exposed to rHE4 in the media. Deposition of laminin-332, the heterotrimer comprising LAMC2 and LAMB3 proteins, was increased in OVCAR8 cells treated with rHE4 or conditioned media from HE4-overexpressing cells. Enzymatic activity of matriptase, a serine protease that cleaves laminin-332 and contributes to its pro-migratory functional activity, was enhanced by rHE4 treatment *in vitro*. Proteomic analysis revealed activation of focal adhesion kinase signaling in OVCAR8 cells treated with conditioned media from HE4-overexpressing cells. Focal adhesions were increased in cells treated with rHE4 in the presence of fibronectin. These results indicate a direct role for HE4 in mediating malignant properties of ovarian cancer cells and validate the need for HE4-targeted therapies that will suppress activation of oncogenic transcriptional activation and signaling cascades.

## Introduction

Epithelial ovarian cancer (EOC) is a highly deadly disease owing to the fact that peritoneal metastasis presents early on in 70% of patients (1, 2). Metastasis most commonly occurs by the transcoelomic route (3), which is a complex process involving epithelial-to-mesenchymal transition (EMT), anoikis resistance, spheroid formation, and homing, attachment, and growth of the malignant cells in a new area. Multiple factors play a role in mediating the above listed steps, including proteases, extracellular matrix components, transmembrane molecules, integrins, chemokines and their receptors, metabolic factors, pro-angiogenic factors, microRNAs, and immune factors (4). Although some progress has been made in identifying potential new treatment targets, novel tumor-enriched targets are desperately needed to allow for more effective treatments with fewer side effects.

Human epididymis protein 4 (HE4) is a secreted glycoprotein and a member of the whey acidic protein (WAP) domain-containing family of anti-proteases (5). It has received much attention in recent years due to its diagnostic and prognostic abilities for EOC. The FDA-approved Risk of Ovarian Malignancy Algorithm (ROMA), which uses HE4 serum levels along with CA125 and menopausal status to detect and monitor ovarian cancer, demonstrates improved sensitivity and specificity over the Risk of Malignancy Index (RMI) that uses CA125, pelvic sonography, and menopausal status (6). Serum HE4 levels predict ovarian cancer with fewer instances of false positives in the case of benign gynecological conditions, as compared to CA125 (7). Since the development of ROMA, studies by us and others have focused on the functional effects of HE4 in ovarian cancer, including its effect on chemotherapy resistance, anti-estrogen resistance, invasion, and migration (8–17).

Several studies have pointed to a role for HE4 in invasion and migration in diverse ovarian cancer cell lines (10, 13, 16), and one study found that HE4 overexpression promoted adhesion of SKOV3 cells onto fibronectin (13). Clinically, HE4 levels were higher in tissues with lymph node metastases (16), and HE4 has also been linked to myometrial invasion in endometrial cancer (18–21). Herein, we expand upon the current literature indicating a role for HE4 in promoting metastatic properties, including invasion, migration, and adhesion. We furthermore present a genome-wide analysis of HE4-mediated transcriptional regulation underlying these effects. Finally, because we have previously shown that HE4 interacts with growth factor signaling cascades (12) and promotes activation of extracellular signal-regulated kinase (ERK) (8), we also sought to elucidate HE4-mediated protein kinase signaling that may contribute to the observed phenotypes.

## Materials and Methods

### Cell Culture and Treatments

OVCAR8 cells and their derivative lines were cultured in Dulbecco-Modified Eagle’s Medium with 10% fetal bovine serum and 1% penicillin/streptomycin, in a humidified incubator at 37°C/5% CO_2_. OVCAR8 cells stably expressing null-vector control plasmid (OVCAR8-NV) and HE4 overexpression plasmid (OVCAR8-C5) were established as previously described, with OVCAR8-C5 cells secreting HE4 levels > 800 pM (12). OVCAR8 wild-type (WT) cells were treated with 20 nM human recombinant HE4 (rHE4; MyBioSource, MBS355616) or 50% conditioned media from OVCAR8-C5 cells for various lengths of time, as indicated.

### Microarray

Subconfluent OVCAR8-WT cells were treated with rHE4 in triplicate for 6 h, and RNA was isolated by Trizol extraction/LiCl precipitation. Subconfluent OVCAR8-NV and OVCAR8-C5 cells were collected in triplicate and RNA isolated in the same manner. Purity was determined by NanoDrop 2000 (Thermo Scientific), and RNA integrity was measured by Bioanalyzer (Agilent 2100) before microarray analysis (Affymetrix HuGene-1_0-st-v1) of 150 ng starting material at the Brown University Genomics Core Facility. Raw intensity values were converted to CELS files, and Transcriptome Analysis Console (TAC) Software was used to generate fold changes and ANOVA *p*-values. ANOVA *p*-values < 0.05 were considered significant. The top 15 genes in either direction were determined for OVCAR8-WT cells (untreated/rHE4-treated) and OVCAR8-NV/OVCAR8-C5 cells. Genes that were changed in both OVCAR8-WT (untreated/rHE4-treated) and OVCAR8-NV/OVCAR8-C5 comparisons with fold change greater than 1.5 in either direction were determined.

### Database for Annotation, Visualization, and Integrated Discovery (DAVID) Gene Ontology Analysis

The DAVID v6.7 (22, 23) was used to identify the top four enriched annotation terms among genes differentially expressed (1.5-fold in either direction, *p* < 0.05) between OVCAR8-WT (untreated/rHE4-treated) and OVCAR8-NV/OVCAR8-C5, as well as among overlapping genes in these two comparisons. Default DAVID parameters were employed as follows:

Kappa Similarity: Similarity Term Overlap—3; Similarity Threshold—0.5
Classification: Initial Group Membership—3; Final Group Membership—3; Multiple Linkage Threshold—0.5
Enrichment Threshold: EASE—1.0
Stringency: Medium

### Quantitative Reverse Transcription Polymerase Chain Reaction (qRT-PCR)

RNA was isolated by Trizol extraction/LiCl precipitation. Total RNA (500 ng) was reverse transcribed into cDNA using an iScript cDNA Synthesis Kit (Bio-Rad, 1708890) according to the manufacturer’s protocol. For microarray validation, the same RNA samples were used. 1 µL cDNA reaction, 2 µL each of 5 µM custom forward and reverse primers (Invitrogen, Sino Biological Inc.) or 1 µM forward and reverse-validated primers (http://realtimeprimers.com), 10 µL SYBR Green (Applied Biosciences [ABI], 4367659), and 5 µL RNAse-free water were added to each well of a 96-well plate for qRT-PCR analysis. Plates were run on an ABI 7500 Fast Real-Time PCR System, and data were analyzed using the ΔΔCt method. Relative expression levels were normalized to 18S rRNA to correct for equivalent total RNA levels. Validated *LAMC2* and *LAMB3* primers were purchased from http://realtimeprimers.com. Validated *SERPINB2* primers were purchased from Sino Biological Inc. (HP100614). Custom primer sequences (Invitrogen) are as follows:

GREM1—F-GGGAGCCCTGCATGTGAC
GREM1—R-GAAGCGGTTGATGATGGTG
TNC—F-AAGCGGGGAATGTTGGGATAG
TNC—R-TAGTCTCCTTTCCACCCCTC
18S rRNA—F-CCGCGGTTCTATTTTGTTGG
18S rRNA—R-GGCGCTCCCTCTTAATCATG

### Western Blot

Protein was extracted in Cell Lysis Buffer (Cell Signaling, 9803) with 1 mM PMSF, and concentrations were determined by DC Protein Assay (Bio-Rad Laboratories, 5000116). Equal amounts of protein boiled with Novex Sample Reducing Agent (Life Technologies, NP009) and NuPAGE LDS sample buffer (Thermo Fisher Scientific, NP0007) were loaded into a 4–12% gradient NuPAGE Novex Bis-Tris gel [Life Technologies, NP0321BOX (mini) and WG1402BX10 (midi)]. Protein was transferred by semi-dry transfer to methanol-activated 0.2 µm PVDF membranes (Bio-Rad, 162-0177) at 0.12–0.2 A for 1 h. Blocking was performed in 5% milk in phosphate-buffered saline with 0.05% Tween 20 (PBS-T) for 30 min at room temperature. Membranes were incubated in primary antibody in 5% milk in PBS-T overnight at 4°C and then in secondary antibody in 5% milk in PBS-T for 1 h at room temperature, with PBS-T washes in between. HRP-tagged secondary antibodies were detected by Amersham ECL Prime Western Blot Detection System (GE Healthcare, RPN2232). Blots were imaged directly in a Bio-Rad ChemiDoc MP Imaging System. GAPDH was used as a loading control. Original images can be seen in Figure S1 in Supplementary Material. Antibodies and dilutions used are as follows:

LAMC2 (Santa Cruz, sc-28330, 1:200)
LAMB3 (Santa Cruz, sc-135968, 1:200)
GAPDH (Cell Signaling, 2118, 1:2,000)

### Densitometry

Densitometry analysis of Western blots was performed using Image J. Blots were analyzed in eight-bit TIFF format with the “analyze gel” function. Band densities were normalized to GAPDH or the appropriate total protein for phosphoproteins. The lowest value was set to 1 for plotted graphs.

### Phosphoproteomics

OVCAR8-WT cells were treated with 50% OVCAR8-C5 conditioned media for 48 h or left untreated. Protein was collected using lysis buffer provided in the Proteome Profiler Human Phospho-Kinase Array Kit (R&D Systems, ARY003B). The manufacturer’s instructions for the kit were followed, and membranes were developed in a Bio-Rad ChemiDoc MP Imaging System. Image J was used to perform background subtraction and determine spot density.

### Invasion Assays

For one replicate of the invasion assays, a Cytoselect 24-Well Cell Invasion Kit (8 μm, Colorimetric, Cell Biolabs, CBA-110) was used according to the manufacturer’s instructions. After overnight starvation, OVCAR8 cells (1 × 10^5^/well) were plated in triplicate in serum-free media in cell culture inserts in the presence or absence of 20 nM rHE4. Media containing 10% FBS were inserted into the lower chamber. After 24 h, media were aspirated from the insert, and the top side of the insert was cleaned with a cotton swab. The insert was then crystal violet stained and washed, and the stained cells were extracted. Extraction solution (150 µL) from each sample was then transferred to a 96-well plate and OD measured at 550 nm. For the following two experimental replicates, 8 µm Transwell Permeable Supports Coated with Cultrex BME (Corning Inc., 3458) were used, with crystal violet staining and acetic acid extraction.

### Adhesion Assays

Cytoselect 48-Well Cell Adhesion Assay Kit (Fibronectin-Coated, Colorimetric; Cell Biolabs, CBA-050) was used to determine the effect of 20 nM rHE4 treatment on adhesion of OVCAR8-WT cells. Cells were plated in triplicate in serum-free media at 1 × 10^5^/well with or without 20 nM rHE4 in the assay plate for 2 h. Media were aspirated, and cells were stained, washed, and extracted. Extraction solution (150 µL) from each sample was transferred to a 96-well plate and OD read at 550 nm.

### Haptotaxis Assays

Haptotaxis assays were conducted using Transwell plates (6.5 µm thickness, 8 µm pores; Corning Inc., 3422). The lower surfaces of the Transwell membranes were coated by adding 500 µL of serum-free PRF-DMEM/F12 containing 2 µg/mL human fibronectin to the lower reservoir overnight. OVCAR8 cells in serum-free PRF-DMEM/F12 were seeded into the upper reservoirs of the Transwell inserts in the presence or absence of 20 nM HE4 and allowed to migrate overnight. Non-migrated cells were removed from the upper surface of the membrane using a Q-tip, and the cells attached to the lower surface were stained with 0.4% crystal violet in sodium borate buffer, pH 9.2 for 5 min, and then washed 2× in water. Crystal violet was eluted from the cells using acetic acid and measured spectrophotometrically at 550 nm.

### Matriptase Assay

A SensoLyte Rh110 Matriptase Activity Kit (AnaSpec, 72241) was used according to the manufacturer’s protocol. Various doses of rHE4 (10, 20, and 40 nM) were tested, and fluorescent readouts (490/520 nm) were performed after 30 min incubation at 37°C. Leupeptin was used as an inhibitor control. Average fluorescence from the substrate control wells was subtracted from the rHE4 test control wells, and the remainder was subtracted from the values obtained for the rHE4-treated wells.

### Immunofluorescence

To visualize focal adhesions, OVCAR8 cell plated on glass coverslips were starved in phenol red-free, serum-free media for 48 h. Starved cells were treated with 1 µg/mL rat fibronectin (Millipore, 341668) in the absence or presence of 20 nM HE4 for 2 h at 37°C. Cells were then washed with PBS and fixed in 4% paraformaldehyde for 10 m and permeabilized in 0.1% Triton X-100 for 5 m. Cells were blocked in 4% normal horse serum in PBS for 30 m and then incubated with phosphotyrosine clone 4G10 antibody (Millipore, 05-321) in 4% normal horse serum (1:500) for 1 h at room temperature. Coverslips were washed in PBS, and cell-associated antibodies were detected using DyLight 594 anti-mouse secondary (Vector Laboratories, DI-2594) diluted in 4% normal horse serum (1:1,000) for 45 min at room temperature. After staining, coverslips were washed and mounted on glass slides in Vectashield with DAPI (Vector Laboratories, H-1200). Imaging was performed on a Zeiss Axio Imager M1 using associated AxioVision software. Quantification was performed in Image J by measuring mean gray values of 4–6 fields per replicate after background subtraction (rolling ball radius = 50 pixels, sliding paraboloid). Results are the average of three independent experiments with 1–3 replicates each.

For laminin-332 staining, OVCAR8-WT cells were seeded onto glass coverslips and treated the next day with 20 nM rHE4 for 5 h. Cells were washed with PBS, fixed in 4% paraformaldehyde for 10 min, and permeabilized in 0.1% Triton X-100 for 5 m. Blocking was performed in 4% normal horse serum for 30 min, and the cells were incubated with anti-laminin-332 antibody (Abcam, ab14509) diluted in 4% normal horse serum (1:200) overnight at 4°C. The following day, the coverslips were washed in PBS and incubated in DyLight 488 anti-rabbit secondary (Vector Laboratories, DI-1488) diluted in 4% normal horse serum (1:1,000) for 45 min at room temperature. Coverslips were mounted using Vectashield with DAPI (Vector Laboratories, H-1200). Imaging and quantitative analysis were performed by an experienced technician at the RI Hospital Core Digital Imaging Facility. Results are the average of three independent experiments with three replicates each.

### Statistics

Where statistics are shown, *n* ≥ 3 independent experiments with biological replicates ≥3, and *p*-values were determined by unpaired one-tailed Student’s *t*-test. Transcriptome Analysis Console (TAC) software (Affymetrix) was used to generate fold changes and ANOVA *p*-values for microarray data.

## Results

### Recombinant HE4 Promotes Invasion, Haptotaxis, and Adhesion of Ovarian Cancer Cells

**Figure 1:**
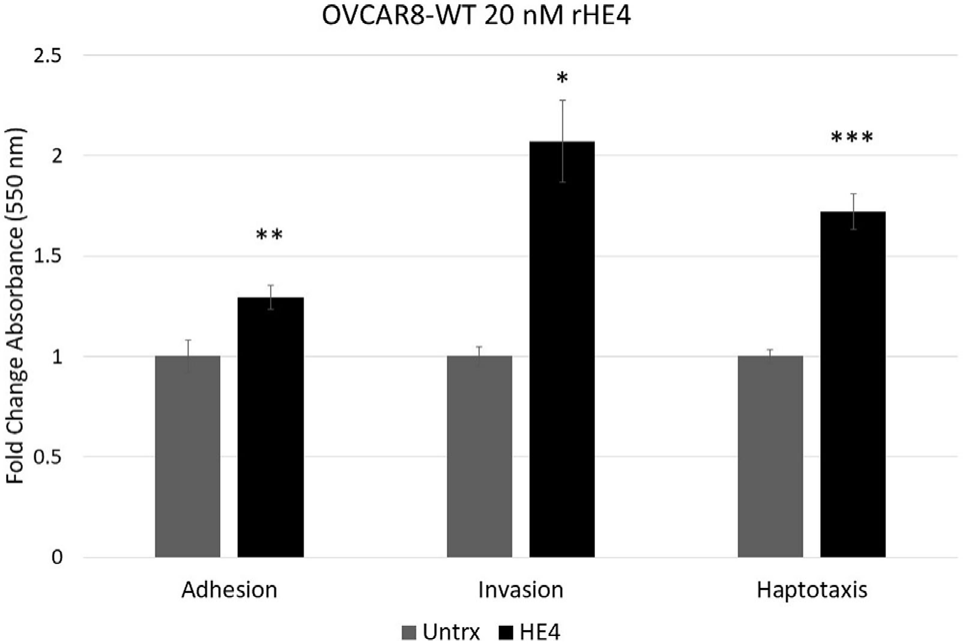
Human epididymis protein 4 (HE4) promotes invasion, adhesion onto a fibronectin substrate, and haptotaxis toward a fibronectin substrate OVCAR8 invasion in response to recombinant HE4 (rHE4) treatment for 24 h was evaluated using Transwell plates. Adhesion in response to 2 h rHE4 treatment was evaluated using fibronectin-coated plates. HE4-mediated haptotaxis toward a fibronectin substrate (24 h treatment) was evaluated using Transwell plates. Error bars indicate standard error of the mean of three biological replicates. *p < 0.05, **p < 0.005, and ***p < 0.0005 versus controls.

To evaluate the effect of HE4 on malignant characteristics of OVCAR8 cells, we performed assays to evaluate invasion, adhesion, and migration. When invasion capacity was evaluated, OVCAR8-WT cells treated with rHE4 were found to have a 2.07-fold greater invasion capacity than untreated cells (*p* = 0.010788). Treatment with rHE4 also increased adhesion of cells to a fibronectin matrix by 1.29-fold (*p* = 0.002257). Interestingly, adhesion onto other substrates—collagen I and IV, laminin I, and fibrinogen—was not altered (data not shown), revealing the specificity of HE4’s effect on adhesion to fibronectin. We then tested the effect of rHE4 treatment of OVCAR8 cells on haptotaxis toward a fibronectin substrate. Treatment with rHE4 increased haptotaxis of the cells by 1.72-fold (*p* = 0.000378) (Figure 1). Collectively, our results demonstrate that HE4 has a direct effect on metastatic properties of ovarian cancer cells.

### HE4 Promotes Expression of Extracellular Matrix-Related Transcripts

To investigate the effect of recombinant HE4 treatment on transcriptional regulation in OVCAR8 ovarian cancer cells, we treated subconfluent cells with 20 nM rHE4 for 6 h. Simultaneously, we collected RNA from OVCAR8 cells stably overexpressing HE4 (C5) or a null vector plasmid (NV). We have previously shown that stable overexpression of HE4 in OVCAR8-C5 cells produces HE4 levels in conditioned media of >800 pM (12). Serial dilution revealed an HE4 concentration of 504 pM in media diluted 1:10 and 51 pM in media diluted 1:100 (data not shown). Total RNA was submitted for microarray analysis comparing OVCAR8-NV to OVCAR8-C5 and OVCAR8-WT untreated cells to OVCAR8-WT rHE4-treated cells. The top 15 annotated, protein-coding genes significantly changed in either direction were identified, as shown in Table 1. The complete results are available through ArrayExpress under the accession number E-MTAB-6366.

**Table 1:** Transcripts differentially expressed between OVCAR8-NV/OVCAR8-C5 (A), and OVCAR8-wild-type (WT)-untreated/OVCAR8-WT-recombinant human epididymis protein 4 (rHE4) treated (B) cells.

Gene symbol	Description	Fold change (NV/C5)	ANOVA *p*-value
**(A)**			
PUS3	Pseudouridylate synthase 3	3.95	0.006158
ZFHX4	Zinc finger homeobox 4	3.37	0.018182
RIF1	Replication timing regulatory factor 1	3.13	0.018553
NIPAL2	NIPA-like domain containing 2	3.12	0.015462
ALDH1L2	Aldehyde dehydrogenase 1 family, member L2	3.11	0.031764
SENP1	SUMO1/sentrin-specific peptidase 1	3.07	0.046433
SCD	Stearoyl-CoA desaturase (delta-9-desaturase)	2.92	0.000165
ALDH2	Aldehyde dehydrogenase 2 family (mitochondrial)	2.85	0.003031
CBX5	Chromobox homolog 5	2.78	0.017494
WDR11	WD repeat domain 11	2.72	0.03599
EPM2AIP1	EPM2A (laforin) interacting protein 1	2.67	0.020269
SOS2	Son of sevenless homolog 2 (*Drosophila*)	2.65	0.015816
ANKRD36C	Ankyrin repeat domain 36C	2.61	0.01709
STARD4	StAR-related lipid transfer (START) domain containing 4	2.59	0.007734
FMNL2	Formin-like 2	2.42	0.046658
PPP2R2C	Protein phosphatase 2, regulatory subunit B, gamma	−2.88	0.000237
CPT1A	Carnitine palmitoyltransferase 1A (liver)	−2.89	0.00653
SH2D4A	SH2 domain containing 4A	−2.98	0.000202
SRGN	Serglycin	−3.07	0.025255
LOXL2	Lysyl oxidase-like 2	−3.19	0.001994
PDE10A	Phosphodiesterase 10A	−3.23	0.010738
FAM167A	Family with sequence similarity 167, member A	−3.4	0.000337
RBM23	RNA binding motif protein 23	−3.44	0.001084
TFAP2C	Transcription factor AP-2 gamma (activating enhancer binding protein 2 gamma)	−3.61	0.000047
LAMB3	Laminin, beta 3	−3.96	0.000972
LOX	Lysyl oxidase	−5.2	0.000123
IL1B	Interleukin 1, beta	−6.26	0.000154
GREM1	Gremlin 1, DAN family BMP antagonist	−6.9	0.000245
SERPINB2	Serpin peptidase inhibitor, clade B (ovalbumin), member 2; serpin peptidase inhibitor, clade B (ovalbumin), member 10	−11.83	0.000129
PCDHB5	Protocadherin beta 5	−12.55	0.000002

**(B)**			
TXNIP	Thioredoxin interacting protein	3.37	0.002051
TSTD3	Thiosulfate sulfurtransferase (rhodanese)-like domain containing 3; RNA, Ro-associated Y1	2.97	0.0405
ERBB3	v-erb-b2 avian erythroblastic leukemia viral oncogene homolog 3	2.82	0.000181
DDIT4	DNA-damage-inducible transcript 4	2.44	0.001939
PDGFRB	Platelet-derived growth factor receptor, beta polypeptide	2.2	0.004343
ABHD4	Abhydrolase domain containing 4	2.16	0.005399
CFLAR	CASP8 and FADD-like apoptosis regulator	2.14	0.007158
TCP11L2	t-Complex 11, testis-specific-like 2	2.11	0.002729
PNRC1	Proline-rich nuclear receptor coactivator 1	2.1	0.007168
FZD2	Frizzled class receptor 2	2.1	0.006872
CHAC1	ChaC, cation transport regulator homolog 1 (*Escherichia coli*)	2.08	0.004698
YPEL1	Yippee-like 1 (*Drosophila*)	2.06	0.001307
DENND2A	DENN/MADD domain containing 2A	2.06	0.004369
H1F0	H1 histone family, member 0	2.06	0.000124
RNF144B	Ring finger protein 144B	2.04	0.001955
LAMC2	Laminin, gamma 2	−2.98	0.000037
GFPT2	Glutamine-fructose-6-phosphate transaminase 2	−3.01	0.00551
DAW1	Dynein assembly factor with WDR repeat domains 1	−3.22	0.002614
GBP1	Guanylate binding protein 1, interferon-inducible; guanylate binding protein 1, interferon-inducible pseudogene 1; guanylate binding protein 3	−3.3	0.000914
LAMB3	Laminin, beta 3	−3.36	0.00313
IL6	Interleukin 6	−3.66	0.000997
GREM1	Gremlin 1, DAN family BMP antagonist	−3.76	0.00024
NFKB1	Nuclear factor of kappa light polypeptide gene enhancer in B-cells 1	−3.91	0.000125
CLDN1	Claudin 1	−4.02	0.000019
TNFAIP3	Tumor necrosis factor, alpha-induced protein 3	−4.18	0.000029
SERPINB2	Serpin peptidase inhibitor, clade B (ovalbumin), member 2; serpin peptidase inhibitor, clade B (ovalbumin), member 10	−6.16	0.000212
CXCL8	Chemokine (C–X–C motif) ligand 8	−6.78	0.000043
PTX3	Pentraxin 3, long	−7.39	0.000438
TNFRSF9	Tumor necrosis factor receptor superfamily, member 9	−8.27	0.000216
CCL20	Chemokine (C–C motif) ligand 20	−8.49	0.000587

*RNA was isolated from OVCAR8-WT cells that were treated with 20 nM rHE4 for 6 h or left untreated, as well as OVCAR8-NV and OVCAR8-C5 cells (*n* = 3/group)*.

*Affymetrix HuGene-1_0-st-v1 arrays were performed to determine differences in transcription between OVCAR8-NV and OVCAR8-C5 cells*.

*The top 15 annotated, protein-coding genes (*p* < 0.05) in either direction are listed below*.

To narrow down our genes of interest, we then identified all transcripts that were changed ≥1.5-fold in either direction by both HE4 overexpression and rHE4 treatment (Table 2). Six of these genes were regulated in opposite directions (DENN/MADD domain containing 2 A [*DENND2A*], family with sequence similarity 19, member 1A [*FAM19A1*], dehydrogenase/reductase member 3 [*DHRS3*], transgenlin [*TAGLN*], leucine rich repeat interacting protein 1 [*LRRFIP1*], and interleukin 6 [*IL6*]), suggesting that stable overexpression of HE4 has some different effects on transcription than short-term exposure of cells to HE4 protein. While we found a few transcripts that were downregulated by HE4, many upregulated genes were of particular interest because of their involvement with invasion and metastasis in diverse tumor types.

**Table 2:** Transcripts differentially expressed with both human epididymis protein 4 (HE4) overexpression and recombinant HE4 (rHE4) treatment.

Gene	Description	Fold change [wild-type(WT)/rHE4]	*p*-Value	Fold change (NV/C5)	*p*-Value
TXNIP	Thioredoxin-interacting protein	3.37	0.002051	2.19	0.020774
DENND2A	DENN/MADD domain containing 2A	2.06	0.004369	−1.65	0.004878
HCP5	HLA complex P5 (non-protein coding)	1.99	0.00043	2.05	0.011577
SLC7A11	Solute carrier family 7 (anionic amino acid transporter light chain, xc− system), member 11	1.83	0.047507	1.8	0.03044
ZHX2	Zinc fingers and homeoboxes 2	1.76	0.020866	1.63	0.013495
GYPE	Glycophorin E (MNS blood group)	1.65	0.012421	2	0.000986
FAM19A1	Family with sequence similarity 19 [chemokine (C–C motif)-like], member A1	1.6	0.001928	−1.68	0.018979
SRR	Serine racemase	1.6	0.010635	1.81	0.032937
CTH	Cystathionine gamma-lyase	1.58	0.003199	1.55	0.018231
PHF21A	PHD finger protein 21A	1.56	0.029529	1.64	0.016533
OPN3	Opsin 3	1.55	0.001048	1.6	0.003847
DHRS3	Dehydrogenase/reductase (SDR family) member 3	1.55	0.005985	−1.55	0.001151
GDF6	Growth differentiation factor 6	−1.57	0.003831	−1.7	0.019611
PDLIM4	PDZ and LIM domain 4	−1.66	0.024732	−1.59	0.036986
TAGLN	Transgelin	−1.68	0.003717	1.57	0.015488
CREB5	cAMP-responsive element binding protein 5; uncharacterized LOC401317	−1.7	0.00433	−1.65	0.027959
ANGPTL4	Angiopoietin-like 4	−1.72	0.002656	−1.92	0.011665
LOX	Lysyl oxidase	−1.73	0.001689	−5.2	0.000123
BDKRB1	Bradykinin receptor B1	−1.77	0.005153	−1.73	0.020509
PDE10A	Phosphodiesterase 10A	−1.88	0.007142	−3.23	0.010738
AJAP1	Adherens junctions associated protein 1	−1.96	0.000665	−1.71	0.030333
TRPC4	Transient receptor potential cation channel, subfamily C, member 4	−1.99	0.009085	2.24	0.005873
LRRFIP1	Leucine-rich repeat (in FLII)-interacting protein 1	−2.02	0.012928	1.67	0.046273
NPPB	Natriuretic peptide B	−2.12	0.005071	−1.53	0.038528
TNC	Tenascin C	−2.18	0.000167	−1.95	0.000676
FGF5	Fibroblast growth factor 5	−2.64	0.004082	−1.67	0.011438
LAMC2	Laminin, gamma 2	−2.98	0.000037	−2.15	0.024177
LAMB3	Laminin, beta 3	−3.36	0.00313	−3.96	0.000972
IL6	Interleukin 6	−3.66	0.000997	1.79	0.018987
GREM1	Gremlin 1, DAN family BMP antagonist	−3.76	0.00024	−6.9	0.000245
SERPINB2	Serpin peptidase inhibitor, clade B (ovalbumin), member 2; serpin peptidase inhibitor, clade B (ovalbumin), member 10	−6.16	0.000212	−11.83	0.000129

*All transcripts changed ≥1.5-fold in either direction in both OVCAR8-NV/OVCAR8-C5 comparisons, and OVCAR8-WT-untreated/OVCAR8-WT-rHE4-treated are listed below*.

**Figure 2:**
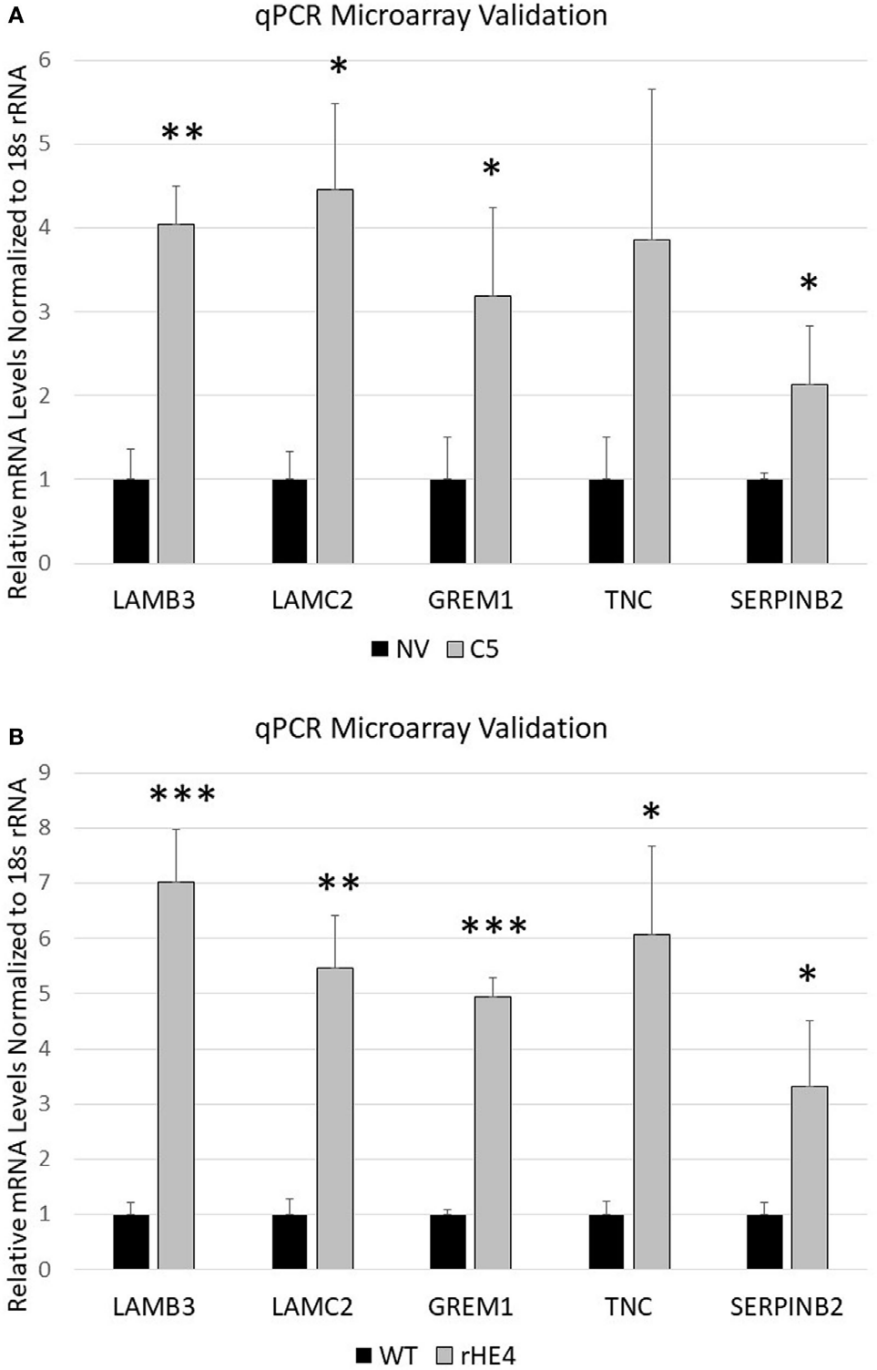
Quantitative reverse transcription polymerase chain reaction validation of microarray *SERPINB2, GREM1, LAMC2, LAMB3*, and *TNC* were selected to validate microarray results in OVCAR8-NV versus OVCAR8-C5 **(A)** and OVCAR8-wild-type (WT) untreated versus recombinant human epididymis protein 4 (rHE4)-treated **(B)**. Error bars represent the SD of three biological replicates, *p < 0.05, **p < 0.005, and ***p < 0.0005 versus controls.

Several genes that were significantly upregulated code for extracellular matrix proteins, such as serpin peptidase inhibitor, member 2 (*SERPINB2*), gremlin 1 (*GREM1*), laminin-β3 (*LAMB3*), laminin-γ2 (*LAMC2*), fibroblast growth factor 5 (*FGF5*), tenascin C (*TNC*), adherens junctions associated protein 1 (*AJAP1*), and growth and differentiation factor 6 (*GDF6*). We validated the upregulation of *SERPINB2, GREM1, LAMB3, LAMC2*, and *TNC* by qRT-PCR (Figures 2A,B). *SERPINB2* was upregulated by 2.13-fold (*p* = 0.024991) and 3.31-fold (*p* = 0.015549) by HE4 stable overexpression and rHE4 treatment, respectively; *LAMB3* was upregulated by 4.04-fold (*p* = 0.0004) and 7.02-fold (*p* = 0.00022), respectively; *LAMC2* was upregulated by 4.45-fold (*p* = 0.002671) and 5.46-fold (*p* = 0.00079), respectively; *GREM1* was upregulated by 3.19-fold (*p* = 0.015549) and 4.94-fold (*p* = 0.0000241), respectively; and *TNC* was upregulated by 3.86-fold (*p* = 0.028017) and 6.06-fold (*p* = 0.002942), respectively.

Gene ontology analysis of the differentially expressed transcripts revealed enrichment of terms related to the extracellular matrix, cell migration, adhesion, and growth (Table 3). We furthermore noted that treatment of OVCAR8-WT cells with rHE4 promoted enrichment of gene terms related to phosphorylation/protein kinase activity, suggesting that addition of exogenous HE4 may have an effect on protein kinase cascades.

**Table 3:** Top 4 Database for Annotation, Visualization, and Integrated Discovery annotation clusters of differentially expressed transcripts.

	Wild-type (WT)/recombinant human epididymis protein 4 (rHE4)		NV/C5		Overlap	
			
	Summary of annotation terms	Enrich-ment score	Summary of annotation terms	Enrich-ment score	Summary of annotation terms	Enrich-ment score
1	Growth factor activity, extracellular space, extracellular region (part), and cytokine activity	4.24	Regulation of growth/cell growth/cell size/cellular component size, negative regulation of growth/cell growth/cell size	2.56	Extracellular region (part)/space	4.84

2	Positive regulation of cell motion/migration/locomotion, cell motility/migration/motion, localization of cell	3.89	Extracellular region (part), extracellular space	2.39	Regulation of growth/cell growth/cell size/cellular component size, negative regulation of growth/cell growth/cell size	2.47

3	(Positive) regulation of cell migration/motion/locomotion, regulation of phosphorylation/phosphorus/phosphate metabolic process, positive regulation of kinase/protein kinase/transferase activity, activation of protein kinase C activity by G-protein-coupled receptor protein signaling pathway, regulation of protein modification process, positive regulation of molecular function, activation of protein kinase activity, and positive regulation of catalytic activity	3.86	Steroid/sterol/cholesterol metabolic process	1.99	Extracellular matrix (part), proteinaceous extracellular matrix, basement membrane, and cell/biological adhesion	2.02

4	Positive regulation of myeloid cell and erythrocyte differentiation, regulation of homeostatic process, negative regulation of cell cycle	2.44	Lipid/sterol/cholesterol homeostasis, lipid/sterol/cholesterol transport, steroid metabolic process, lipid localization/transport, cholesterol efflux, steroid binding	1.92	Regulation of leukocyte migration/cell migration/locomotion/cell motion	1.81

*All transcripts with fold change ≥1.5 (*p* < 0.05) in either direction between OVCAR8-NV/OVCAR8-C5, OVCAR8-WT-untreated/OVCAR8-WT-rHE4-treated, and overlapping transcripts in these two comparisons were analyzed*.

### HE4 Upregulates LAMC2 and LAMB3 Protein in a Time-Dependent Manner and Increases Laminin-332 Levels

**Figure 3:**
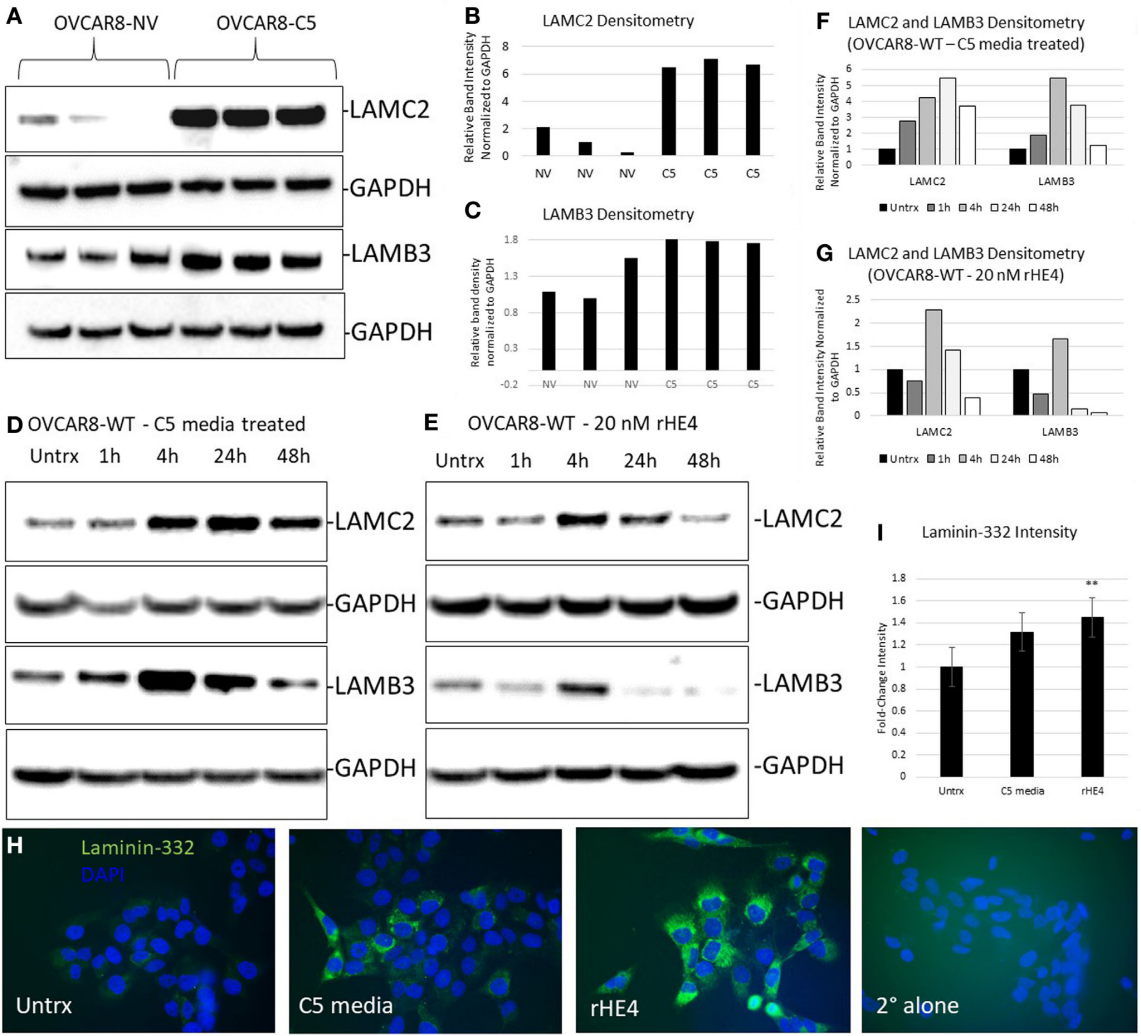
Human epididymis protein 4 (HE4) promotes an increase in laminin-332. **(A)** Protein levels of LAMC2 and LAMB3 in OVCAR8-NV and OVCAR8-C5 cells were determined by Western blot. GAPDH was used as a loading control. **(B,C)** Densitometry analysis of LAMC2 and LAMB3 (normalized to GAPDH) from **(A)**. **(D,E)** Time course analysis of protein levels in OVCAR8-wild-type (WT) cells left untreated or treated with 20 nM recombinant HE4 (rHE4) or 50% conditioned media from OVCAR8-C5 cells. **(F,G)** Densitometry analysis of LAMC2 and LAMB3 (normalized to GAPDH) from Western blot in **(D,E)**. **(H)** OVCAR8 cells were treated with rHE4 or conditioned media from OVCAR8-C5 cells for 5 h, and immunofluorescence staining for laminin-332 was performed. Representative images are shown. Green = laminin-332, blue = DAPI, scale = 40×. **(I)** Intensity was determined for laminin-332. Results are the average of fold change from three independent experiments with three replicates per experiment and four fields for each replicate. Error bars indicate standard error of the mean. **p < 0.005 versus control.

We chose to further investigate HE4’s regulation of LAMC2 and LAMB3, since these two genes code for chains of laminin-332, a secreted heterotrimer that has been well-described to promote aggression and metastatic properties in diverse cancers (24–31). LAMC2 and LAMB3 protein levels were constitutively elevated in OVCAR8-C5 cells compared to OVCAR8-NV (Figures 3A–C), while their levels peaked between 4 and 24 h after treatment with both rHE4 and conditioned media from OVCAR8-C5 cells (Figures 3D–G). Interestingly, C5 media (which has an approximate HE4 concentration of 5 nM) elicited a stronger response than 20 nM rHE4, suggesting that naturally secreted HE4 may be more bioactive than recombinant protein. Collectively, these results reveal a time-dependent effect of exogenous HE4 on LAMC2 and LAMB3 and indicate that stable overexpression of HE4 leads to constitutively high levels of these two proteins.

Next, we performed immunofluorescence analysis of the complete laminin-332 heterotrimer to determine if overexpression of its subunits promotes increased levels of the complete heterotrimer. Laminin-332 staining (mean gray value) was increased by 1.36-fold with C5 media treatment (*p* = 0.069025) and by 1.48-fold with rHE4 treatment (*p* = 0.002926), indicating that elevated levels of LAMC2 and LAMB3 proteins resulted in increased secretion of the laminin-332 heterotrimer (Figures 3H,I).

### HE4 Enhances Enzymatic Activity of Matriptase, a Serine Protease That Cleaves Laminin-332

**Figure 4:**
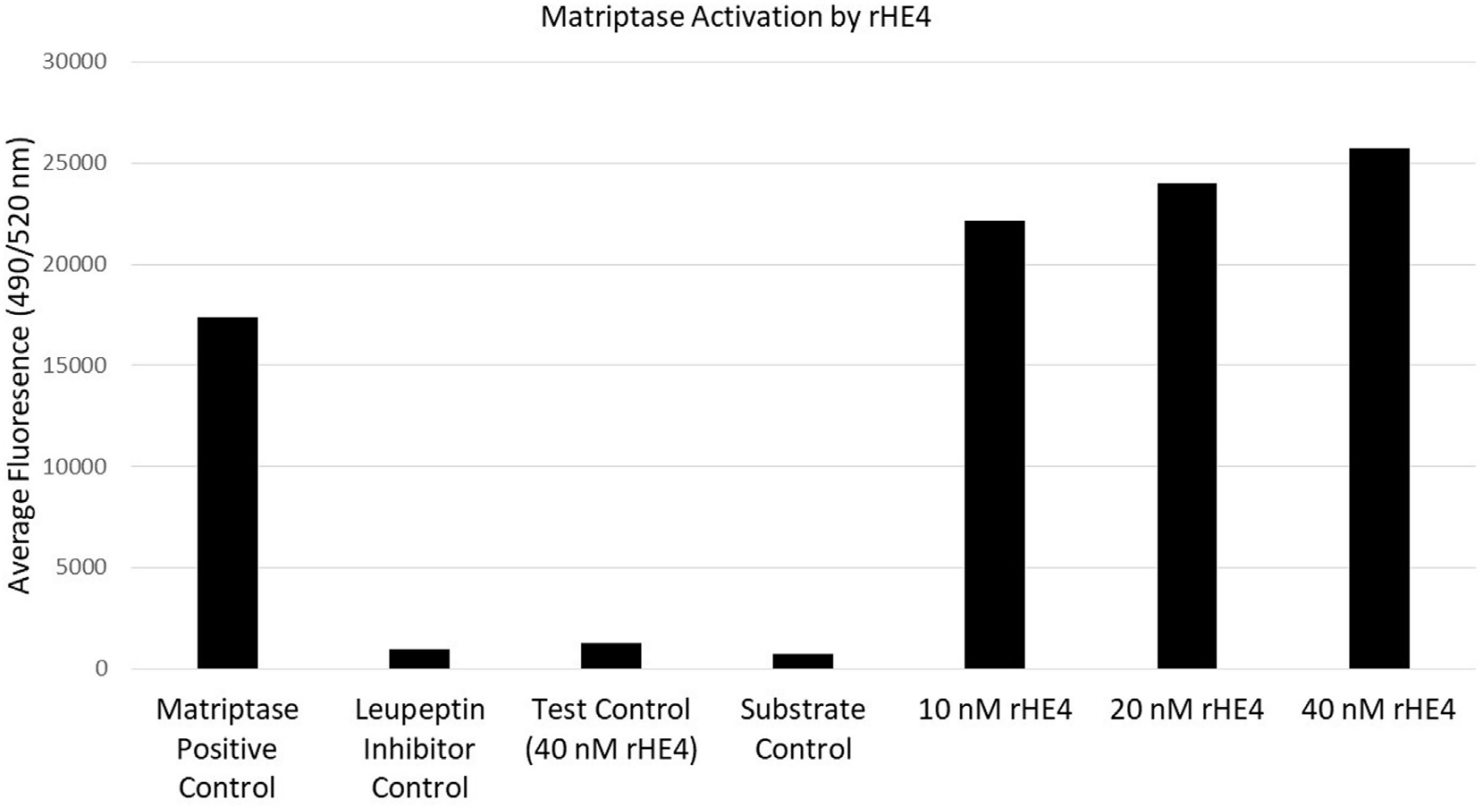
Human epididymis protein 4 (HE4) enhances enzymatic activity of matriptase. The effects of various doses of recombinant HE4 (rHE4) on *in vitro* matriptase activity were evaluated. The experiment was performed 3–5 times per dose, and the results from one representative experiment including all three doses are shown. Leupeptin = matriptase inhibitor.

Since HE4 has been shown to inhibit the activity of multiple proteases (32, 33), we hypothesized that it might also inhibit enzymatic activity of matriptase, a serine protease that is known to proteolytically cleave laminin-332 in its β3 chain. Surprisingly, we observed the opposite to be true. *In vitro* matriptase activity was enhanced by rHE4 in a dose-dependent manner, while the inhibitor control (leupeptin) almost entirely obliterated matriptase activity (Figure 4). When the results of 3–5 separate experiments per dose were averaged, we saw an average 1.26-fold (±0.534075) increase in activity by 10 nM rHE4 (*p* = 0.04139), 1.32-fold (±0.489149) by 20 nM rHE4 (*p* = 0.000798), and 1.63-fold (±0.61337) by 40 nM rHE4 (*p* = 0.026596) (data not shown). Although these results are in opposition to the presumed role of rHE4 as a protease inhibitor, they indicate that HE4 not only upregulates laminin-332 levels but may also contribute to regulation of its proteolytic processing, which is known to promote migration in prostate cancer cells (34).

### HE4 Promotes Activation of Focal Adhesion Kinase (FAK) Signaling

**Figure 5:**
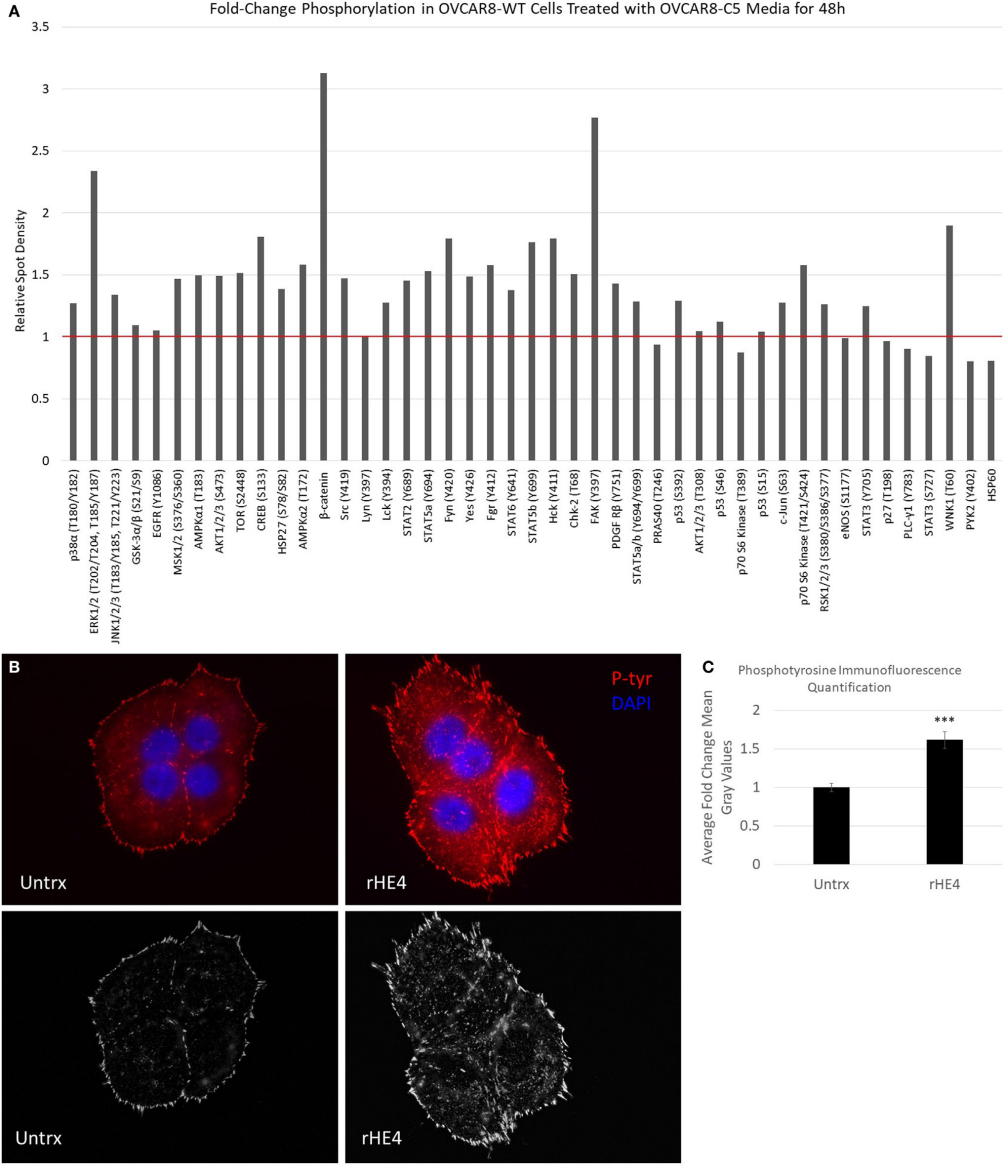
Human epididymis protein 4 (HE4) promotes increased formation of focal adhesions in the presence of fibronectin. **(A)** Proteome Profiler Human Phospho-Kinase Array (Cell Biolabs) was used to compare levels of protein phosphorylation in OVCAR8-wild-type (WT) cells (untrx) versus OVCAR8-WT cells treated with 50% conditioned media from HE4-overexpressing OVCAR8-C5 cells for 48 h. Red bar delineates proteins with increased phosphorylation. **(B) Upper panel**: focal adhesions in untreated versus recombinant HE4 (rHE4)-treated OVCAR8-WT cells. Focal adhesions are indicated by phosphotyrosine staining (red). Blue = DAPI. Scale = 100×. **(B)** Lower panel: focal adhesions are shown with background subtraction performed in Image J (rolling ball radius = 50 pixels). **(C)** Quantification of phosphotyrosine intensity. Results are the average of fold change from three independent experiments, with 1–3 replicates per experiment and 4–6 fields per replicate. Error bars indicate standard error of the mean. ***p < 0.0005 versus control.

Next, we pursued the hypothesis that HE4 promotes activation of diverse kinase signaling pathways. The activation of MAPK signaling by HE4 has been well documented by us and others (8, 10, 13, 14), but we suspected that activation of other signaling pathways is also stimulated by HE4. We treated OVCAR8-WT cells with conditioned media from OVCAR8-C5 cells for 48 h and analyzed phosphorylation of an array of kinases and their target proteins using a Human Phospho-Kinase Array (R&D Systems; Figure 5A). Upregulation of several phosphoproteins was observed, including phospho-ERK (2.34-fold), as we have previously reported (8). Of note, we also found β-catenin and phospho-FAK (Y397) upregulated by 3.13- and 2.76-fold, respectively. β-Catenin is an intracellular signal transducer of the Wnt-signaling pathway, which has roles in cell–cell adhesion (35), and FAK is a Src-family tyrosine kinase that promotes cell adhesion to the extracellular matrix (36). Furthermore, multiple other members of the Src-family of tyrosine kinases, including phospho-Fgr (Y412), phospho-Fyn (Y420), phospho-Hck (Y411), phospho-Src (Y419), and phospho-Yes (Y426), were all upregulated by around 1.5-fold or more in response to exogenous HE4 exposure.

To confirm the effects of HE4-mediated activation of FAK signaling, we stimulated starved OVCAR8-WT cells with rat fibronectin in the presence or absence of rHE4 for 2 h and examined focal adhesions by phosphotyrosine staining. Cells that were treated with rHE4 had 1.61-fold more focal adhesions than untreated cells (*p* = 0.0000593), indicating that HE4 phosphorylation of FAK at Y397 promotes subsequent formation of focal adhesions (Figures 5B,C).

## Discussion

Several relevant gene transcripts were affected by HE4 overexpression or treatment as indicated by our microarray results. *SERPINB2* was the foremost upregulated transcript by stable HE4 overexpression and rHE4 treatment. Traditionally, SERPINB2, a secreted glycoprotein that inhibits tPA and urokinase, has been described as a tumor suppressor that inhibits tumor growth and metastasis, whereas SERPINB1 promotes tumor progression (37). This description of SERPINB2 function appears to contradict the effect of HE4 on metastatic properties; thus, the connection between these two proteins and the significance of *SERPINB2* upregulation by HE4 is an area for further investigation. The secretion of SERPINB2 protein could be evaluated, since one study found that the unsecreted, intracellular protein had a different role than the secreted protein and protected against TNFα-induced apoptosis (38).

*GREM1*, which was also significantly upregulated by HE4 stable overexpression or rHE4 treatment, is overexpressed in a wide variety of cancers, including uterine cervical, lung, ovary, kidney, breast, colon, pancreas, and sarcoma (39), and has been shown to promote metastatic properties. GREM1 tissue expression is associated with EMT and coordinates migration at the cancer invasion front in colon cancer (40, 41). Xu et al. also found overexpression of GREM1 in peritoneal metastatic ovarian cancers in comparison to primary tumors (42), indicating the importance of this protein in ovarian cancer progression.

*LAMC2, LAMB3*, and *LAMA2* genes encode for the γ2, β3, and α3 chains that comprise the cross-shaped heterotrimer laminin-332, a secreted glycoprotein that is a major component of the basement membrane of epithelial tissues. Laminin-332 serves as a ligand of various transmembrane receptors including α3β1 and α6β4 integrins. Importantly, abnormal expression and high levels of laminin-332 have been shown to promote invasion in colon, breast, and skin cancers (30). Specific chains of the laminin-332 heterotrimer have been correlated with various aspects of tumor aggression as well. For example, LAMB3 was correlated with chemoresistance and poor prognosis in Stage III colorectal cancer (43). Of particular interest, one study found that expression of another laminin, *LAMB2*, was mediated by HE4. The authors reported that HE4 promoted invasion and migration in CaOV3 cells *via* HE4 binding with Annexin-V, and downregulation of the HE4 gene suppressed the expression of *MKNK2* and *LAMB2*. Treatment with exogenous HE4 protein reversed this effect (15). In a recent report, laminin-332 was found to promote EMT and correlate with poor prognosis in lung cancer. The authors found that collagen XVII stabilized laminin-332 and caused activation of the FAK pathway (31), which is relevant to our current results showing upregulation of laminin-332 and FAK activation in response to HE4 exposure.

It is likely that the tyrosine phosphorylation of FAK that we noted in cells treated with exogenous HE4 was a result of rapid upregulation of laminin components following HE4 treatment. Laminin binding to integrin receptors mediates FAK activation (31, 44–46), which in turn activates other Src family kinases to regulate cell survival, apoptosis, proliferation, adhesion, migration, and invasion (36). Activated FAK (Y397) is localized at focal adhesions, which are points of contact between the ECM and the actin cytoskeleton (36). Thus, our results showing increased numbers of focal adhesions with rHE4 treatment are in agreement with the observed upregulation of FAK phosphorylation. Inhibition of this major signaling kinase is a promising cancer therapeutic; however, tumor-specific targeting of FAK remains a barrier to this approach (36). It is possible that inhibiting a regulator of FAK signaling such as HE4, which is selectively upregulated in ovarian tumor tissue, may overcome this problem. Furthermore, evidence thus far suggests that HE4 regulates multiple signaling pathways and aspects of tumor progression, making it a more attractive therapeutic target.

We also wanted to gain insight into possible mechanisms of HE4 regulation of laminin-332. Although we presumed some regulation to occur *via* signaling and regulation of gene expression of laminin-332 components, we wondered if HE4’s role as a putative protease inhibitor could contribute to its effect on laminin-332 levels. Matriptase is a serine protease that is known to cleave laminin-332 in the β3 chain, thereby promoting the effects of laminin-332 on motility (34). The role of matriptase in ovarian cancer is still being defined, but studies show that its expression is upregulated at early stages of the disease, while it is lost in Stage III/IV tumors (47, 48). While this may seem to indicate that matriptase expression is beneficial in ovarian cancer, this is not a foregone conclusion. One study also found that advanced stage tumors that do express matriptase are less likely to coexpress its inhibitor, HAI-1 (47). Another study found that the high-metastatic human ovarian cancer cell line HO-8910 had higher levels of matriptase than the homologous HO-8910 cell line, and knockdown of matriptase effectively inhibited the cells’ invasion and migration abilities (49). These results suggest that unchecked matriptase activity may play a role in the aggression of a percentage of late-stage tumors. Therefore, if HE4 enhances matriptase activity, this may potentially contribute to increased invasion and migration.

We wondered if HE4 might affect matriptase activity, since it has been shown to act as a cross-class protease inhibitor (32, 33) and belongs to the “four-disulfide core” family that encompasses several extracellular protease inhibitors (50). Our results revealed that recombinant HE4 actually enhances the activity of matriptase in a dose-dependent manner, which shows for the first time that HE4 can promote activity of at least one serine protease. It is possible that the increased matriptase activity may work in concert with upregulation of laminin-332 by HE4 to promote laminin-332 functions affecting migration, invasion, or adhesion. Further exploration of this hypothesis and clarification of how HE4 increases matriptase activity are underway.

Our results give way to many other questions that will need to be investigated in future studies. First, what is the mechanism of the rapid upregulation of LAMC2 and LAMB3 protein expression by HE4? Although it has become clear that HE4 affects many aspects of ovarian cancer progression, the molecular mechanisms behind these effects are not yet clear. It is possible that secreted HE4 binds to cellular receptors to affect intracellular signaling, since we have previously noted HE4 interaction with EGFR (12). However, a specific HE4 receptor has yet to be identified. It is also possible that intracellular HE4 has different functions than extracellular, which is suggested by our data showing differential regulation of some genes in HE4-overexpressing cells and rHE4-treated cells. The possibility of a nuclear role for HE4 may also exist, given our previous results showing nuclear translocation of HE4 upon treatment of cells with various growth factors (12).

Other questions raised by our results remain to be answered as well. Does activation of FAK by HE4 primarily occur through laminin signaling, or does HE4 affect signaling through diverse mechanisms? Finally, the cause of the specificity of HE4 adhesion to fibronectin over other substrates could also be examined. Furthermore, while we tested an array of potential substrates, there are several types of each substrate. It is possible that HE4 may promote adhesion onto one or more of these other forms of extracellular matrix components.

Our results are in agreement with other reported studies on the role of HE4 in promoting metastatic properties, indicating a consistency to the effect of HE4 in diverse cell types. However, it is likely there are also cell-type specific differences in precisely how HE4 promotes invasion, migration, and adhesion. Therefore, it would be interesting to determine the clinical relevance of our findings by determining if laminin proteins or FAK activation correlate with high levels of HE4 in ovarian cancer tissue.

## Conclusion

Together, our results support recent research connecting HE4 to increased migratory and invasive capacities, as well as establish a role for HE4 in adhesion to a fibronectin substrate. We have found that HE4 affects ECM protein expression and cell-signaling promoting invasion, haptotaxis, and adhesion of ovarian cancer cells. Furthermore, we have shown for the first time that HE4 enhances the enzymatic activity of the serine protease matriptase, which not only has implications for its role in tumor metastasis but also for the establishment of HE4’s molecular function. Such results firmly establish HE4 as an important protein not just in the clinical diagnosis and management of EOC but also in its mechanistic basis.

## Availability of Data Sets

The microarray data set supporting the conclusions of this article is included within the supplementary materials, and also is available through ArrayExpress under accession number E-MTAB-6366.

## Author Contributions

JR, HG, RM, PD, and NY designed experiments. JR and HG executed all experiments with assistance from MK, NJ, and MO. NY developed null vector and HE4-overexpressing stable clones. CS performed the microarrays and assisted with data analysis. JR prepared this manuscript. All the authors reviewed and approved the manuscript.

## Conflict of Interest Statement

The authors declare that the research was conducted in the absence of any commercial or financial relationships that could be construed as a potential conflict of interest.

## Supplementary Material

Figure S1 Original uncropped Western blot images.

## Abbreviations

C5, clone 5; EOC, epithelial ovarian cancer; EGFR, epidermal growth factor receptor; ERK, extracellular signal-regulated kinase; HGSC, high-grade serous ovarian cancer; MAPK, mitogen-activated protein kinase; NV, null vector plasmid; qRT-PCR, quantitative reverse transcription polymerase chain reaction; rHE4, recombinant human epididymis protein 4; RMI, Risk of Malignancy Index; ROMA, Risk of Ovarian Malignancy Algorithm; WAP, whey acidic protein; WT, wild-type.
