# Supplementary figures and images for: Human Epididymis Protein 4 Promotes Events Associated with Metastatic Ovarian Cancer via Regulation of the Extracelluar Matrix
Source: Front Oncol. 2018 Jan 22;7:332. doi: 10.3389/fonc.2017.00332 (PMC5786890; doi:10.3389/fonc.2017.00332)

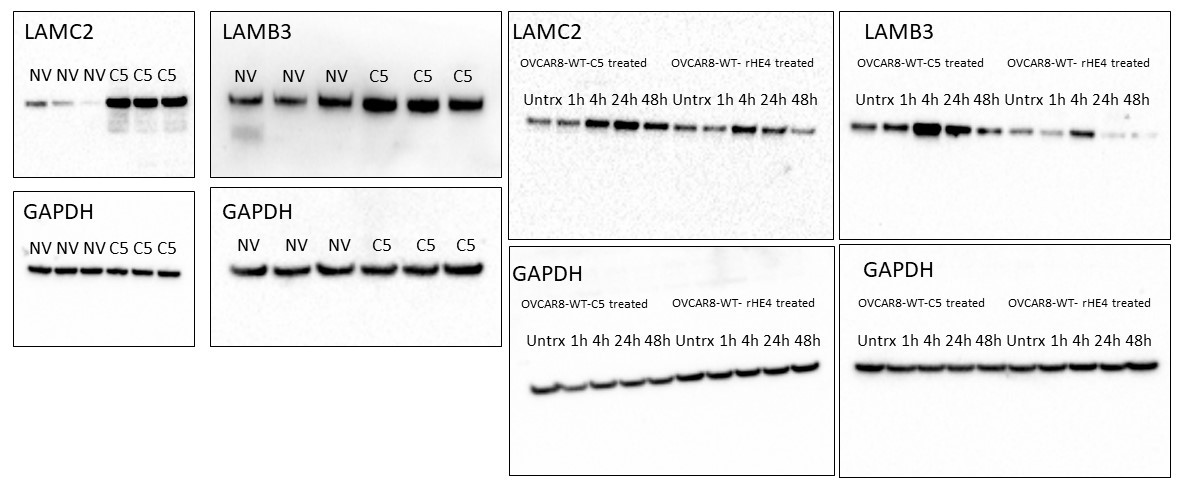

Supplement: Figure S1 — Original uncropped Western blot images. [file Image_1.jpeg]
